# The association between types of regular primary care and hospitalization among people with and without multimorbidity: A household survey on 25,780 Chinese

**DOI:** 10.1038/srep29758

**Published:** 2016-07-20

**Authors:** Roger Y. Chung, Stewart W. Mercer, Benjamin H. K. Yip, Stephanie W. C. Chan, Francisco T. T. Lai, Harry H. X. Wang, Martin C. S. Wong, Carmen K. M. Wong, Regina W. S. Sit, Eng-Kiong Yeoh, Samuel Y. S. Wong

**Affiliations:** 1 School of Public Health and Primary Care, Faculty of Medicine, The Chinese University of Hong Kong, Hong Kong; 2 General Practice and Primary Care, Institute of Health and Wellbeing, University of Glasgow, Scotland, UK; 3 School of Public Health, Sun Yat-Sen University, Guangzhou, P.R. China

## Abstract

Using data collected from 25,780 Hong Kong citizens in a household survey, this study aimed to investigate the association between having regular source of primary care and hospitalization amongst people with and without multimorbidity (two or more chronic conditions). Potential interaction effects of regular primary care with multimorbidity were also examined. Results revealed a significant association between having regular source of primary care from General Practitioners and reduced hospitalization amongst respondents with multimorbidity (RR = 0.772; 95% CI = 0.667–0.894), adjusting for other potential confounding factors (i.e., socio-demographic factors and medical insurance and benefits). In contrast, having regular Specialist care was significantly associated with increased risk of hospitalization among both people with multimorbidity (RR = 1.619; 95% CI = 1.256–2.087) and without multimorbidity (RR = 1.981; 95% CI = 1.246–3.149), adjusting for potential confounders. A dose-response relationship between the number of chronic diseases and hospitalization was also observed, regardless of whether participants had regular source of primary care or not; relative risks and predicted probabilities for hospitalization were generally greater for those without regular source of primary care. Further studies are warranted to explore the role of healthcare system, informatics, organizational and practice-related factors on healthcare and functional outcomes.

World Health Organization (WHO) has estimated that by 2050, 22% of the world’s population will be aged 60 or over^1^. With this aging trend, there is a global epidemic of long-term chronic conditions^2^, and the related long-term care expenditure has been increasing around the world^3^. Hong Kong is no exception to this trend – it has been projected that the long-term care expenditure for elderly will increase from 1.4% in 2004 to 4.9% of GDP by 2036^4^. In face of this increasing spending on healthcare, healthcare system needs to be more efficient than ever before. Primary care has been shown to be associated with many beneficial outcomes, including improved health outcomes^5^, lower mortality^6^,^7^, better preventive care^8^, more equitable distribution of health^6^ and more cost-effective care^9^. In particular, regular source of primary care is found to be associated with reduced hospitalizations^6^,^10^, which require great amount of professional and technological resources, and incur high medical costs.

With this epidemic of long-term chronic conditions, there is also an increasing number of people living with two or more chronic conditions, also known as multimorbidity. Multimorbidity is well-established to be associated with various adverse outcomes, including higher risk of premature mortality than single disease, longer hospital stay, greater health service use and healthcare costs^11^,^12^,^13^,^14^,^15^,^16^. Nevertheless, despite greater emphasis on the management of chronic conditions in healthcare systems across the world, the delivery of care worldwide is generally built upon the management and treatment of single diseases^17^, and evidence on the effectiveness of interventions to improve outcomes of multimorbid patients is still limited. No studies have specifically explored the relationship between regular source of primary care and hospitalization compared across people with and without multimorbidity.

Therefore in this study, we utilized a large population-representative household dataset in Hong Kong to investigate the association of regular source of primary care with number of hospital admissions among people with and without multimorbidity. We hypothesized that regular source of primary care would be associated with reduced hospitalizations, especially among people with multimorbidity, but the association may be less apparent for people without multimorbidity. In Hong Kong, primary care is not administered solely by Family Physicians or General Practitioners, and can be administered by physicians of other specialties. The difference between “General Practitioners” and “Family Physicians” is that GPs can be non-Family Medicine specialists, while Family Medicine specialists are recognized as Fellows of the Hong Kong Academy of Medicine who are required to complete additional vocational training and professional examinations. Currently, as in January 2016, there are only 446 Family Medicine specialists in Hong Kong. Also, since Hong Kong does not have a mandatory registry for doctors who provide primary care services, some patients would bypass the traditional primary care doctors (i.e., GPs and Family Physicians) to directly consult specialists of other specialties, who would provide primary care to these patients if necessary. Therefore, as a sub-analysis, we also tested the interaction effect between the source of primary care received and multimorbidity in influencing hospitalizations.

## Methods

### Data collection and study population

The Census and Statistics Department (C&SD) of the Hong Kong SAR Government has conducted a series of territory-wide cross-sectional survey, the Thematic Household Survey (THS), to collect data on different social topics since 1999. The data in the present study was obtained from the round of THS conducted from October 2011 to January 2012, which collected data on various health-related topics including health conditions, medical insurance coverage, health care utilization and hospitalization. In addition, information on socio-demographic characteristics was also obtained.

The THS covered land-based population of Hong Kong but excluded institutionalized residents, persons living on board vessels, foreign domestic helpers and hotel transients. Altogether the survey covered around 95% of the Hong Kong resident population. The sampling units are permanent quarters in built-up areas and segments in non-built-up areas. For each sampled area segment, all units of quarter within the segment were first enumerated. Then, all households within the quarter and all persons excluding live-in domestic helpers within the households were enumerated. Each household and each resident had an equal selection probability. A total of 13,411 households were sampled, and among them, 10,065 households had been successfully interviewed face-to-face with structured questionnaires, giving an overall response rate of 75%^18^. Altogether, 29,187 persons were interviewed. After excluding subjects aged below 15 who were not requested to answer questions on health-related topics, a total of 25,780 subjects were included for analyses in the present study. For more information on the THS 2011–12, please refer to the published report by the Census and Statistics Department of the Hong Kong Government^18^. The questionnaire of the THS can also be requested from the Census and Statistics Department of the Hong Kong Government.

### Independent variables

#### Regular source of primary care

Although Hong Kong does not have a mandatory primary care registry, primary care was nevertheless given by practitioners to their patients. In the present study, regular source of primary care is defined as having a usually visited doctor in Hong Kong, whom the subject would first consult when he/she is sick or need preventive health care services. Those who answered “Yes” to this question were then further asked to indicate the source of their usually visited doctor, including “General Practitioner,” “Family Physician,” and a range of “other specialist doctors”. As such, GPs and Family Physicians were examined as two separate items in subsequent analyses in order to explore potential differences in their impacts on hospital admission. In addition, participants who selected other specialists as their usually visited doctors were grouped under the category “other specialists”. Please refer to the last paragraph of Introduction section for more detailed descriptions of the three sources of primary care in Hong Kong. These three categories were compared among each other and against those having “no usually visited doctor,” in other words, no regular source of primary care”.

#### Socio-demographic characteristics

Information on age, sex, marital status, migrant status, highest education attained, average monthly household income and employment status was obtained.

#### Medical insurance and benefits

Information on subjects’ medical insurance and benefits was obtained. Medical insurance and benefits included those provided by current or previous employer of the subjects or the subjects’ household members, as well as family and personal medical insurance policies and medical riders that covered the subjects.

#### Multimorbidity

We obtained self-reported information on chronic conditions as diagnosed by their Western medicine practitioners. These conditions include cancer, diabetes, hypertension, heart diseases, stroke, asthma, hypercholesterolemia, disease of the blood, immune disease, mental disorder, disease of the nervous system, disease of the eye, disease of the ear/nose/throat, disease of the circulatory system, respiratory diseases, stomach and intestinal disease, liver disease, skin disease, musculoskeletal and connective tissue diseases, kidney or reproductive system diseases and complications of previous injury (please refer to Appendix 1 for details). The number of chronic diseases of each subject was then derived. Subjects with two or more chronic diseases were regarded as those with multimorbidity, as commonly defined^19^,^20^,^21^.

### Outcome variables

#### Number of hospital admissions

Subjects who were admitted to private or public hospital in the past 12 months at the time of their survey interviews between October 2011 and January 2012 were identified as having hospital admission. The number of hospital admissions in the past 12 months was used as the outcome.

### Statistical analyses

Using negative binomial regression, relative risks and corresponding 95% confidence intervals (95% CI) were calculated to examine the association between having regular source of primary care and the number of hospital admissions, adjusting for other potential confounding factors (i.e., socio-demographic factors and medical insurance and benefits). To ensure robustness of results in face of possible clustering effects by household, generalized estimating equations (GEE) were used for estimations. First, we conducted stratified analyses to examine whether the association between the presence of regular source of care and the number of hospital admissions differed among those with and without multimorbidity. In particular, we compared the effects of having no regular source of primary care, having regular primary care from GPs, having regular primary care from Family Physicians, and having regular care from other specialists. Second, using GEE based on negative binomial regression, we examined whether an interaction effect existed between the source of regular source of primary care and multimorbidity (in terms of number of chronic diseases) to influence the number of hospital admissions. Moreover, from the interaction model, we estimated the predicted probabilities of hospitalization by number of chronic diseases among subjects in the four categories of regular care. We used the standard negative binomial regression to estimate the mean predicted probabilities and their 95% CI.

GEE analyses were conducted with STATA 13, while all other analyses were conducted using R.

## Results

### Socio-demographic characteristics

The socio-demographic characteristics of the study population are presented in Table 1. Of the 25,780 subjects included in this study, 47.8% were male and 52.2% were female. The subjects were evenly distributed across different age groups from 15–24 to 65 or above (years), with proportions ranging from 15.3% to 20.6% out of the sample. More than half of the subjects were married (56.5%), had a secondary education (58.5%), were born in Hong Kong (66.9%), and were employed (54.9%). The average monthly household income distribution was more skewed towards the lower end. Slightly more than half of the subjects had no medical benefits or insurance (53.5%). Regarding their source of primary care, the majority of the participants had no usually visited doctor (65.9%), while 30.6% had a usually visited GP. Only a very small proportion of respondents had a family physician or other specialist doctor that they usually visited (2.8% and 0.7% respectively). Additionally, 30.5% of the subjects had one or more chronic diseases, and 13.4% had multimorbidity, i.e., two or more chronic diseases. Finally, most subjects had not been hospitalized in the past 12 months at the time of their interviews (94.4%), 4.3% were admitted once, and 1.3% were admitted more than once.

### Regular source of primary care and hospitalization

As exemplified in Table 2, for subjects without multimorbidity (i.e., with no or one chronic disease only), there is no statistically significant association between having regular source of primary care and number of hospital admissions, after adjustments for socio-demographic factors and medical insurance and benefits coverage, with the exception of the regular source of primary care being other specialists. Additionally, Table 2 also indicates that higher number of hospital admissions was associated with 65 years old or above, being divorced/separated or widowed, being migrants, non-employment, having household income of $10,000 to $19,999 as well as having medical insurance or benefits among subjects without multimorbidity. In particular, it was interesting to note the sizable effect of marital status, whereby respondents who were divorced/separated from their spouse were four times more likely to have higher hospital admissions compared to those who were single (RR = 4.107; 95% CI = 3.150–5.355). On the contrary, respondents with education level of secondary schools had reduced hospital admissions.

For subjects with multimorbidity, after adjustments for other potential confounding factors (i.e. socio-demographic factors and medical insurance and benefits), having regular source of primary care from GP was associated with reduced number of hospital admissions (RR = 0.772; 95% CI = 0.667–0.894), while the opposite is observed for those with regular primary care from other specialists (RR = 1.619; 95% CI = 1.256–2.087). Moreover, higher number of hospital admissions was also associated with being male and non-employment. On the contrary, respondents with an education level of primary and secondary schools had reduced hospital admission.

### Interaction effect of source of primary care and number of chronic diseases on hospitalization

**Figure 1:**
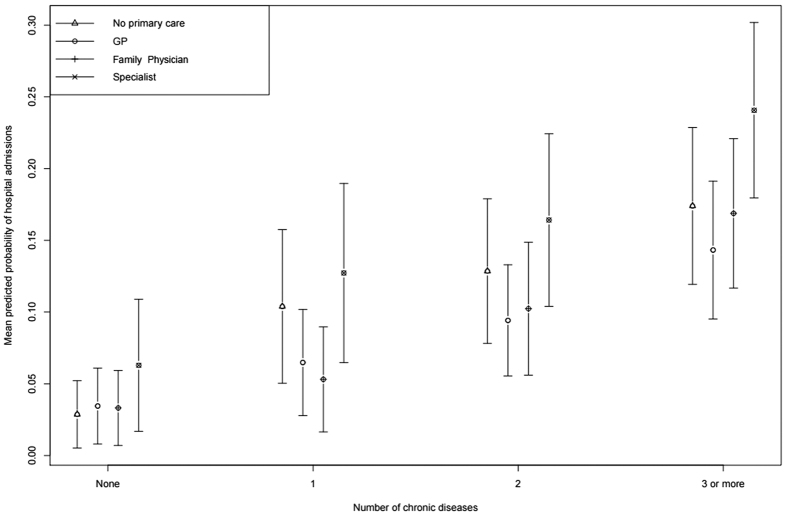
Predicted probability of hospital admissions by number of chronic diseases (0, 1, 2 and 3 or more) among patients aged 15 or above. Circles indicate the mean values, and error bars indicate 95% confidence intervals.

Table 3 (Fig. 1) shows the interaction effects of the source of primary care and number of chronic diseases on hospitalization in relative risks (in predicted probabilities). Findings from our interaction analysis revealed a dose-response effect between the number of chronic diseases and probability of hospital admission, whereby the probability is notably higher for those with greater number of diseases. Also importantly, those without any form of regular primary care have observably higher probability of hospital admission compared to those with regular GPs or Family Physicians, regardless of the number of chronic diseases. However, those with regular primary care from other specialist doctors significantly show an even higher probability of hospital admission than those with regular GPs or Family Physicians, as well as those with no regular source of primary care.

## Discussion

### Summary of findings

In this large population representative study, we have shown that having a regular source of primary care from GP is associated with reduced hospital admissions among people with multimorbidity, while having a regular source of primary care from other specialists is associated with increased hospital admissions among people with or without multimorbidity. Also, the risks of being admitted to hospitals were generally higher for those without a regular source of primary care and those with other specialists as regular source of primary care, as compared to those with GPs and Family Physicians as their regular source of primary care, with other specialists having the highest risk. As expected, we have also noticed a dose-response relationship between the number of chronic diseases and number of hospital admissions, regardless of whether there was a regular source of primary care or not.

### Explanations

Our stratified analysis showed that compared to those without regular primary care, respondents with regular primary care from GPs had reduced hospital admission. An obvious explanation for this is that primary care in the form of GPs (i.e., the provision of generalist care) provides better continuity of care, patient-centeredness and coordination that may reduce the chance of hospitalization. On the other hand, it is also possible for people with multimorbidity but no regular source of primary care to monitor their conditions, they may seek medical attention/care from a variety of doctors of varying specialties, which may result in care being fragmented and uncoordinated^22^. The lack of holistic primary care among these patients may lead to poorly managed chronic conditions and increase their possibility of being admitted to hospital. Thus, compared to multimorbid patients with regular GPs visits, multimorbid patients with no regular source of primary care had higher hospitalization. Moreover, this association was observed amongst people with multimorbidity, but not for those without, thus demonstrating the significance of a regular source of primary care particularly amongst people with multimorbidity. This is also reasonable because people without multimorbidity may need much less care, primary or hospitalization regardless, than those with multimorbidity at the first place^15^.

However, it was observed that although regular primary care from GP was significantly associated with reduced hospital admission, regular care from Family Physician did not have the same impact, whether multimorbid or not. One potential explanation for this phenomenon may be because of the much smaller sample size that reflects much lower utilization of Family Physicians (n = 568) than GPs (n = 6,814). This is reasonable because this reflects the reality that there was a much lower number of Family Physicians than GPs in Hong Kong. As such, many of the citizens may be less familiar with the concept of “Family Physician,” possibly resulting in a lower self-reported utilization that may not generate readily observable associations. Nonetheless, additional analysis (data not shown) with regular primary care from GP and Family Physicians as a combined single predictor also revealed that having regular primary care in general (regardless of whether it was from GPs or Family Physicians) was a significant protective factor of hospital admission amongst people with multimorbidity (RR = 0.752; 95% CI = 0.633–0.895). Hence, this further supports our finding of the importance of primary care in reducing hospital admission.

On the contrary, it was observed that those with a usually visited doctor of other specialties had significantly increased hospital admission among both multimorbid and non-multimorbid people. The effect of other specialists on hospitalization was further exemplified in the interaction analysis, where the probability of hospital admission was consistently highest across all number of chronic diseases for people with other specialists as their regular source of care. There are two plausible explanations. First, respondents who visit specialists are likely to be those with more severe conditions that could not be easily or adequately controlled in a primary care setting and thus demand more advanced care. Second, it is also possible that specialists have a greater tendency to admit their patients into hospital compared to General Practitioners. This is consistent with the finding from a previous study where people with a regular family physician were less likely to utilize more expensive accident and emergency and in-patient services^23^. Finally, our interaction analysis using number of chronic diseases instead of the dichotomous multimorbidity status further showed that the relative risks of hospitalization were consistently greater for those without regular source of primary care than those with regular source of care from GPs and Family Physicians among subjects with one or more chronic diseases. This implies that primary care may serve as the gate-keeping function in the healthcare system by reducing the number of hospital admissions that may be unnecessary or avoidable. Patients without a regular source of primary care may over-utilize the hospital in-patient services in cases of sickness; and this may be particularly true in Hong Kong where hospital stays are heavily subsidized by the government. Again, this is consistent with the finding from a previous study where people with a regular family physician were less likely to utilize accident and emergency and in-patient services^23^. On the contrary, among subjects without any chronic disease, the association was reversed, whereby those *with* regular source of primary care showed a greater risk of hospitalization than those *without*. This observation is not surprising as those without any chronic disease and do not have regular primary care may simply be healthy individuals who do not require regular or frequent medical attention, and hence, are less likely to be admitted into hospital for acute conditions or other diseases. Nevertheless, this observation is not of great clinical significance because the difference in predicted probabilities for hospitalization between subjects with and without regular source of primary care was very small.

### Limitations

It is important that we interpret our findings with caution. First, we have only employed a cross-sectional design; hence, causal inferences cannot be drawn from the findings. Second, all medical conditions were self-reported and there could have been under- or over- estimation of medical conditions. While previous similar studies have shown that self-reported medical conditions can be a valid way to measure morbidity in the community^23^,^24^, other studies have shown the otherwise^25^. Therefore, this is a limitation of the present study. Third, data on severity of illness may need to be adjusted in the analysis, because patients receiving primary care from specialists may tend to have greater severity of illness than those receiving care from general practitioners and family physicians. However, this data was not available in the survey, and thus should be incorporated in further investigations in the future. Fourth, since the types of usually visited doctors were self-reported by the respondents, there may potentially be slight misclassifications on the source of primary care. Fifth, the definition of multimorbidity can vary in terms of the number of diseases included in the study as well as the number of these together that constitute to be having multimorbidity. Thus, results may vary depending on the definition used. The conventional definition of multimorbidity with 2+ disease entities can be measured using different definitions of disease entity with as few as 12 prevalent chronic conditions. As shown in Appendix 1, the THS had covered 18 chronic disease entities. However, other recent research had indicated that the 2+ definition may lack specificity, especially for the older population^26^, and suggested an operational definition of multimorbidity using 3+ disease entities, which however requires more measurement conformity and all chronic conditions, not only a selection of conditions^27^. While our present study did not focus only on the elderly, multimorbidity defined as 3+ can be considered in future studies that focus on the elderly. Last, the prevalence estimates of individual chronic diseases and multimorbidity are relatively low compared to other international studies^27^,^28^,^29^,^30^. This may be due to our relatively young sample – i.e., the prevalence of multimorbidity is 44.7% for the elderly aged 65 or above, which is much higher than 13.4% for the entire surveyed population. Nevertheless, the elderly prevalence was still lower than those reported in international literature. This was very possibly due to our healthier sample selected from the households. Thus, we need to be cautious of the interpretation of our findings.

### Public health significance

Although previous studies have demonstrated the association of the presence of regular source of care or longitudinal continuity of care with better health outcomes, few of these have particularly looked into the patient populations with multimorbidity. This is of importance because the presence of a regular source of care or longitudinal continuity of care is closely associated with relational continuity of care^31^, which has been shown to be highly valued by people with multimorbidity^31^,^32^. In addition, although longitudinal continuity of care is important and desired by people with multiple chronic conditions, they are less likely to receive it^13^. Furthermore, another recent local study^33^ has found that less advantaged people tend to have higher risks of multimorbidity and they are also the ones who tend to have poorer primary care experience. All these call for more urgent action to address this issue.

## Conclusion

This study demonstrated the possible importance of primary care given by general practitioners in reducing hospitalizations among people with multiple chronic diseases, thereby potentially reducing the consumption of resources and expenditure generated from higher utilization of emergency and in-patient services that tend to be more expensive. This can better inform future healthcare planning and policy in consolidating the role of regular primary care especially among aging populations living with increasing number of chronic diseases. Nevertheless, future prospective study will be needed to further delineate the causal relationship between having a regular source of primary care and hospitalizations in this population. Moreover, since there is a lack of studies that focused on the care of people with multimorbidity, more studies are needed to explore the role of health system, healthcare informatics, organizational and practice related factors in their impacts on health care and functional outcomes as well as healthcare costs in the care of people with multiple chronic conditions.

## Additional Information

**How to cite this article**: Chung, R. Y. *et al*. The association between types of regular primary care and hospitalization among people with and without multimorbidity. A household survey on 25,780 Chinese. *Sci. Rep.* **6**, 29758; doi: 10.1038/srep29758 (2016).

## Supplementary Material

Supplementary Information

## Figures and Tables

**Table 1:** Socio-demographic characteristics of the study population (aged 15 or above).

Variables	No. of respondents (N, %)
**Total**	25780 (100%)
Sex	
Male	12316 (47.8%)
Female	13464 (52.2%)
Age (years)	
15–24	3967 (15.4%)
25–34	3973 (15.4%)
35–44	4383 (17.0%)
45–54	5317 (20.6%)
55–64	3936 (15.3%)
65 or above	4204 (16.3%)
Marital status	
Single	8534 (33.1%)
Married	14556 (56.5%)
Divorced/Separated	938 (3.6%)
Widowed	1752 (6.8%)
Highest education level achieved	
Kindergarten or below	1460 (5.7%)
Primary	4573 (17.7%)
Secondary	15157 (58.8%)
Tertiary or above	4590 (17.8%)
Migrant status	
Born in Hong Kong	17235 (66.9%)
Mainland China/Macau (<7 years)	807 (3.1%)
Mainland China/Macau (≥7 years)	7065 (27.4%)
Others (<7 years)	155 (0.6%)
Others (≥7 years)	518 (2.0%)
Employed	
Yes	14161 (54.9%)
No	11619 (45.1%)
Average monthly household income ($HK; $1US = $7.8HK)	
<10,000	4372 (17.0%)
10,000–19,999	6179 (24.0%)
20,000–29,999	5638 (21.9%)
30,000–39,999	3856 (15.0%)
40,000–49,999	2259 (8.8%)
>=50000	3476 (13.5%)
Medical insurance & benefits	
Yes	11985 (46.5%)
No	13795 (53.5%)
Chronic disease	
0	17905 (69.5%)
1	4406 (17.1%)
2	1859 (7.2%)
3 or more	1610 (6.2%)
Regular source of primary care	
No	16998 (65.9%)
General Practitioner	7895 (30.6%)
Family Physician	715 (2.8%)
Other Specialists	172 (0.7%)
No. of hospital admissions	
0	24339 (94.4%)
1	1101 (4.3%)
2 or more	340 (1.3%)

**Table 2:** Relative risks with 95% confidence intervals (95% CI) of factors associated with hospital admission in the past 12 months among age 15 or above, stratified by (a) those without multimorbidity (No or 1 chronic disease only) and (b) those with multimorbidity (2 or more chronic diseases) using generalized estimating equations based on negative binomial regression.

Variables	(a) Without multimorbidity		(b) With multimorbidity	
	RR	95% CI	RR	95% CI
Sex				
Female*	1.000		1.000	
Male	0.959	(0.856–1.075)	1.461	(1.286–1.660)^++^
Age				
15–24*	1.000		1.000	
25–34	1.721	(1.274–2.324)^++^	0.713	(0.323–1.572)
35–44	1.428	(1.035–1.972)^+^	0.621	(0.330–1.169)
45–54	1.468	(1.063–2.028)^+^	1.048	(0.630–1.742)
55–64	1.587	(1.138–2.213)^+^	0.922	(0.559–1.519)
65 or above	2.575	(1.837–3.610)^++^	0.746	(0.449–1.240)
Marital Status				
Single*	1.000		1.000	
Married	1.996	(1.624–2.453)^++^	0.917	(0.706–1.191)
Divorced/Separated	4.107	(3.150–5.355)^++^	0.941	(0.654–1.352)
Widowed	2.173	(1.627–2.903)^++^	1.058	(0.791–1.416)
Educational Attainment				
Kindergarten or below*	1.000		1.000	
Primary	0.927	(0.741–1.160)	0.835	(0.716–0.974)^+^
Secondary	0.783	(0.620–0.989)^+^	0.717	(0.597–0.860)^++^
Tertiary or above	1.065	(0.816–1.389)	0.792	(0.608–1.031)
Migrant Status				
Born in Hong Kong*	1.000		1.000	
Mainland China/Macau (≥7 years)	1.224	(1.081–1.386)^++^	1.123	(0.985–1.279)
Mainland China/Macau (<7 years)	1.459	(1.110–1.917)^+^	1.578	(0.926–2.688)
Others (≥7 years)	1.285	(0.925–1.786)	1.214	(0.845–1.746)
Others (<7 years)	1.816	(1.113–2.963)^+^		NA (n = 3)
Employment Status				
Employed*	1.000		1.000	
Non-employment	1.361	(1.183–1.566)^++^	2.033	(1.636–2.528)^++^
Household Income				
<10,000*	1.000		1.000	
10,000–19,999	1.275	(1.080–1.504)^+^	1.166	(1.002–1.356)^+^
20,000–29,999	0.983	(0.815–1.185)	1.138	(0.946–1.368)
30,000–39,999	0.830	(0.665–1.036)	1.117	(0.903–1.382)
40,000–49,999	0.947	(0.739–1.215)	1.054	(0.779–1.424)
>=50000	0.847	(0.673–1.067)	1.193	(0.916–1.553)
Medical benefits and insurance				
Not insured*	1.000		1.000	
Insured	1.635	(1.438–1.859)^++^	0.960	(0.809–1.139)
Regular Source of Primary Care				
No*	1.000		1.000	
General Practitioner	0.963	(0.851–1.090)	0.772	(0.667–0.894)^++^
Family Physician	0.833	(0.570–1.219)	0.997	(0.736–1.351)
Other Specialists	1.981	(1.246–3.149)^+^	1.619	(1.256–2.087)^++^

^+^p < 0.05; ^++^p < 0.001.

^*^Reference category.

**Table 3:** Negative Binomial regression analysis of factors associated with hospital admission in the past 12 months among age 15 or above, with interaction of regular source of primary care and number of chronic diseases.

Combination	RR (95%CI)
No regular source of primary care	
No chronic disease*	1.00
1 chronic disease	3.591 (3.134–4.114)^++^
2 chronic diseases	4.147 (3.536–4.864)^++^
3 or more chronic diseases	5.668 (4.868–6.599)^++^
Regular source of primary care (from GP)	
No chronic disease*	1.210 (1.023–1.432)^+^
1 chronic disease	2.553 (2.129–3.062)^++^
2 chronic diseases	2.862 (2.264–3.617)^++^
3 or more chronic diseases	4.424 (3.627–5.396)^++^
Regular source of primary care (from Family Physician)	
No chronic disease*	1.127 (0.684–1.856)
1 chronic disease	1.837 (1.035–3.26)^+^
2 chronic diseases	3.133 (1.741–5.638)^++^
3 or more chronic diseases	5.589 (3.917–7.974)^++^
Regular source of care (other specialists)	
No chronic disease	2.304 (0.895–5.933)
1 chronic disease	4.457 (2.601–7.639)^++^
2 chronic diseases	5.840 (3.678–9.272)^++^
3 or more chronic diseases	9.682 (7.161–13.092)^++^

Figures are relative risks (95% confidence intervals). ^+^p < 0.05. ^++^p < 0.001.

^*^Reference category: no regular source of primary care and no chronic disease.
