# Supplementary material for: The association between types of regular primary care and hospitalization among people with and without multimorbidity: A household survey on 25,780 Chinese
Source: Sci Rep. 2016 Jul 20;6:29758. doi: 10.1038/srep29758 (PMC4951721; doi:10.1038/srep29758)
Supplement: Supplementary Information [file srep29758-s1.doc]

**The association between types of regular primary care and hospitalization among people with and without multimorbidity. A household survey on 25,780 Chinese**

Article category: Epidemiology

Roger Y. Chung^1^, Stewart W. Mercer ^1,2^, Benjamin H.K. Yip^1^, Stephanie W.C. Chan^1^, Francisco TT Lai^1^, Harry H.X. Wang^1^, Martin C.S. Wong^1^, Carmen K.M. Wong^1^, Regina W.S. Sit^1^, Eng-Kiong Yeoh^1^, Samuel Y.S. Wong^1^*

^1^ School of Public Health and Primary Care, Faculty of Medicine, The Chinese University of Hong Kong, Hong Kong

^2^ General Practice and Primary Care, Institute of Health and Wellbeing, University of Glasgow, Scotland, UK

*** CORRESPONDING AUTHOR:** Prof. S.Y.S. Wong; School of Public Health and Primary Care, Faculty of Medicine/The Chinese University of Hong Kong/4/F School of Public Health, Prince of Wales Hospital, Shatin, New Territories/Hong Kong SAR/ [yeungshanwong@cuhk.edu.hk](mailto:yeungshanwong@cuhk.edu.hk)

| **Appendix 1 – List of the chronic diseases included in the study** | |
| --- | --- |
| **Metabolic conditions**  High blood pressure  High cholesterol  Diabetes mellitus  **Cancer**  Stomach cancer  Colon cancer / Rectal cancer / Anal cancer and  Anal canal cancer  Liver cancer and intrahepatic biliary cancer  Lung cancer and bronchogenic cancer  Nasopharyngeal cancer (NPC)  Breast cancer  Uterine cancer and ovarian cancer  Cervical cancer  Prostate cancer  Other cancer  **Heart diseases**  Coronary heart disease  Other heart diseases  Stroke  **Diseases of the blood**  Anaemia  Hereditary blood disease (e.g. thalassemia, hemophilia)  **Immune disease**  Disorder involving the immune system (e.g. immune deficiency syndrome)  **Endocrine & metabolic diseases**  Thyroid disease  **Mental disorder**  Depression  Anxiety disorder  Schizophrenia  Dementia  **Diseases of the nervous system**  Epilepsy  Parkinson's disease  **Complications of previous injury**  Complications of previous injury (e.g. loss of function of limb(s)) | **Diseases of the circulatory system**  Diseases of the circulatory system (e.g. varicose veins, hemorrhoids)  **Respiratory diseases**  Asthma  Emphysema, chronic bronchitis, bronchiectasis  Tuberculosis  **Stomach & intestinal disease**  Stomach & intestinal disease (e.g. gastric ulcer)  **Liver disease**  Liver disease (e.g. hepatitis B or C, cirrhosis)  **Skin disease**  Skin disease (e.g. eczema, psoriasis)  **Musculoskeletal and connective tissue diseases**  Arthritis, rheumatism  Low back pain  Gout, high uric acid  Osteoporosis  Systemic lupus erythematosus (SLE)  Rheumatoid Arthritis  **Kidney or reproductive system**  Kidney disease (e.g. kidney failure, nephritis, requiring dialysis)  Disease of the reproductive system (e.g. gynaecological disease, prostate disease, breast problem)  **Diseases of the eye**  Diseases of the eye (e.g. glaucoma, cataract, poor vision, blindness)  **Diseases of the ear/ nose/ throat**  Diseases of the ear/ nose/ throat (e.g. sinusitis, allergic rhinitis, hearing loss, tinnitus) |
